# Sequence-dependent heterochromatin formation in the human malaria parasite *Plasmodium falciparum*

**DOI:** 10.1016/j.heliyon.2023.e19164

**Published:** 2023-08-16

**Authors:** Toshiyuki Mori, Mai Nakashima

**Affiliations:** Department of Molecular Protozoology, Research Institute for Microbial Diseases (RIMD), Osaka University, 3-1 Yamadaoka, Suita, Osaka, 565-0871, Japan

**Keywords:** Heterochromatin, Histone modification, Gene silencing, Malaria parasite, Sexual development

## Abstract

The human malaria parasite *Plasmodium falciparum* represses transcription of the gene encoding AP2-G, which is the master regulator of germ cell differentiation, via heterochromatin condensation following histone H3 lysine 9 trimethylation (H3K9me3). Although H3K9me3-marked heterochromatin is typically constitutive and its establishment depends on the RNA interference (RNAi) pathway in fission yeast centromeres, malaria parasites lack molecular members essential for RNAi. We developed a strategy to assess heterochromatin establishment on artificial chromosomes introduced into *P. falciparum*. We show that a particular DNA sequence in the *AP2-G* promoter is able to induce *de novo* H3K9me3 nucleosome deposition. In addition, we also found that the *AP2-G* promoter contains a distinct element required in maintenance of the repression memory. Thus, we speculate that malaria parasites have evolutionarily acquired a sequence-dependent establishment system of non-constitutive, *i.e.* facultative, H3K9me3-marked heterochromatin.

## 1 Introduction

Eukaryotes have highly-condensed chromosomal segments in their nucleus, called heterochromatin, in which the underlying sequences are silenced due to inaccessibility of transcription machinery. While constitutive heterochromatin is marked with histone H3 lysine 9 di- or trimethylation (H3K9me2/3) and remains compacted throughout lifecycle as a structure constituting centromeric and telomeric regions for example, facultative heterochromatin dynamically changes its structure to control gene expression across lifecycle stages [1]. In fission yeast, fruit flies, flowering plants and mammals, establishment of constitutive heterochromatin depends on transcription and non-coding RNA complexed with proteins that recruit an H3K9 methyltransferase to the template genomic sequences, such as centromeric repeats and transposable elements [[2], [3], [4], [5]]. In fission yeast, the maintenance of the silent state depends on a set of *cis* regulatory elements in the mating-type locus that act independently of heterochromatin establishment [6,7]. On the other hand, establishment of facultative heterochromatin is diverged according to species and cell lineages. In many animals and plants, the facultative heterochromatin is typically established by the Polycomb-repressive complex 2 (PRC2), which is recruited to specified sequence contexts and methylates histone H3 lysine 27 (H3K27), leading to H3K27me3-marked chromatin compaction [8,9]. In fruit flies, the specific sequences Polycomb Response Elements (PREs) function as a “silencer” in which PRC2 and the other related complexes are recruited to repress the downstream gene expression, leading to proper development [8]. In addition, PRE is also required for maintenance of the silent state during cell division, although detailed molecular mechanism is unknown [10,11].

In *P. falciparum*, the causative agent of human malaria, typical constitutive heterochromatin is absent from centromeric regions and H3K9me3-marked heterochromatin contributes to regulation of genes involving sexual development (*AP2-G*) and evasion from the host immune system (*var* gene family encoding surface antigens) [[12], [13], [14], [15], [16], [17]]. In addition, no PRC2-related genes have been reported in malaria parasites. These facts imply that the parasites utilize H3K9me3-marked heterochromatin as facultative, corresponding to H3K27me3-marked one. The transcription factor AP2-G is a master regulator of sexual development and a subpopulation of parasites express AP2-G and differentiate into the male or female germ cells in the blood stage of its life cycle [18]. In asexual cells, the *AP2-G* locus is compacted into H3K9me3-marked heterochromatin [19]. Although several studies have shown interesting traits of epigenetic regulation of the *P. falciparum* genes including *AP2-G* [13,15,[20], [21], [22]], the molecular basis of *de novo* heterochromatin formation is still elusive.

Because malaria parasites lack the transcription-dependent pathway for heterochromatin establishment, such as RNA interference (RNAi) in fission yeast [23], we hypothesized that a specific *cis*-element in the *AP2-G* locus participates in *de novo* deposition of H3K9me3 nucleosomes, similar to the silencer element of fruit flies. To verify this hypothesis, we developed a strategy to detect *cis*-elements that induce *de novo* heterochromatin establishment at the *AP2-G* locus based on the use of artificial chromosomes introduced into cultured *P. falciparum*.

## 2 Materials and methods

### 2.1 *P. falciparum* parasite culture and transfection

All the parasite strains were cultured in O+ erythrocytes at 2% hematocrit under the previously-described condition [24]. The conditions and procedures for transfection followed the previously-reported methods [25].

### 2.2 CRISPR/Cas9 genome editing

All the strains were prepared by co-introduction of sgRNA-expressing artificial chromosome vector and donor DNA fragment into the Cas9-expressing parasite 3D7^cas9^, which was produced in the previous study [25].

For production of *AP2-G* deleted (AP2-del) strain, two oligonucleotide pairs MalP3840/3841 and MalP4031/4032 for sgRNA templates were ligated into psgRNA2_cen vector, as previously described [25]. DNA fragments homologous to up- and downstream (HR1 and 2) of sgRNA-targeting sequences were amplified using MalP4033/4034 and MalP4035/4036 respectively. HR1 and 2 fragments were combined into a donor DNA fragment. To confirm the successful deletion of *AP2-G* locus, diagnostic PCR was performed using MalP4051/4115 and MalP4051/4052 (primers 1/2 and 1/3 in Fig. S1 respectively).

For production of DiCre-expressing strain, an oligonucleotide pair MalP1970/1971 for sgRNA template was ligated into psgRNA1_cen vector, as previously described [25]. *P. berghei elongation factor 1a* (PBANKA_1,133,400) promoter, *FKBP12*, *FRB*, *HSP90* (PF3D7_0708400) terminator and *HSP70ter* were amplified using MalP2278/2279, MalP3249/3250, MalP3252/3253, MalP3254/3255 and MalP3251/2073 respectively, and all combined to produce an expression cassette of DiCre. The DiCre cassette was combined with *CSP* (PF3D7_0304600) locus fragments (HR1 and 2) amplified using MalP2704/3247 and MalP2622/3248 respectively, to produce HR1/DiCre cassette/HR2 fragment as a donor DNA targeting *CSP* locus.

### 2.3 Production of reporter strains

Each of all the reporter constructs was introduced into the AP2-Gdel strain described above.

*hDHFR-PbDHFR* terminator fragment was amplified from psgRNA1_cen vector using MalP3070/3071, and combined with *HSP70* (PF3D7_0818900) promoter amplified using MalP3068/3069, to produce *HSP70pro*-*hDHFR-PbDHFRter* cassette. The resultant product was ligated with *Kpn*I-*Bam*HI fragment containing *P. falciparum* centromere (PfCen), which is derived from the psgRNA1_cen vector, to produce the backbone artificial chromosome vector pRep_HSPpro-hDHFR_cen. *mNG* and *AP2-G* 3′-region fragments were amplified using MalP4039/1717 and MalP4040/4041 respectively, and combined to produce *mNG-AP2-G 3′-rgn* fragment. The resultant product was cloned into the pRep_HSPpro-hDHFR_cen vector after digestion with *Kpn*I and *Xho*I. To modify multicloning sites of the vector, the *mNG* region was replaced with a different *mNG* fragment amplified using MalP4059/4060, and the resultant vector was designated pRep_mNG_AP2-G 3′-rgn_cen.

For GG construct, 5′-region of *AP2-G* (*AP2-G 5′-rgn*) locus was amplified using MalP4122/4123 and ligated into the pRep_mNG_AP2-G 3′-rgn_cen vector after digestion with *Nhe*I and *Sal*I.

For GH construct, *AP2-G 5′-rgn-mNG* fragment was amplified from genomic DNA of GG reporter strain, using MalP4122/1715. The PCR product was combined with *P. berghei HSP70* (PBANKA_0711900) terminator (*PbHSP70ter*) amplified using MalP4216/3091 to produce *AP2-G 5′-rgn-mNG-PbHSP70ter* cassette. The resultant product was ligated with the pRep_mNG_AP2-G 3′-rgn_cen vector after digestion with the *Nhe*I and *Xho*I.

For CG construct, *calmodulin* (PF3D7_1434200) promoter (*Campro*) was amplified using MalP4227/4228, and cloned into the pRep_mNG_AP2-G 3′-rgn_cen vector after digestion with *Nhe*I and *Sal*I.

For CH construct, *Campro-mNG* fragment was amplified from the CG construct vector, using MalP4227/1715. The PCR product and *PbHSP70ter* fragment above were cloned into the pRep_mNG_AP2-G 3′-rgn_cen vector after digestion with the *Nhe*I and *Xho*I.

For Del_I and II constructs, partial *AP2-G* 5′-regions were amplified using MalP4721/4499 and MalP4646/4499 respectively. For Del_III-VI constructs, *AP2-G* 5′-untranslated region (UTR) was amplified using MalP4644/4499. Partial *AP2-G* 5′-regions for Del_III-VI were amplified using MalP4642/4643, MalP4642/4726, MalP4721/4726 and MalP4721/4750 respectively, and each of them was combined with the *AP2-G* 5′-UTR fragment above. Each of the resultant PCR products for Del_I-VI was ligated with the pRep_mNG_AP2-G 3′-rgn_cen vector after digestion with *Nhe*I and *Sal*I.

### 2.4 Conditional sequence deletion assay

All the constructs were introduced into the DiCre-expressing strain described above.

For GGe reporter strain, partial *AP2-G* 5′-regions were amplified using MalP4122/4749, MalP4748/4732 and MalP4731/4499, and combined by overlap PCR. *Nhe*I and *Sal*I.

For GGm reporter strain, partial *AP2-G* 5′-regions were amplified using MalP4710/4970 and MalP4969/4499, and combined by overlap PCR.

Each of the resultant products was ligated with the pRep_mNG_AP2-G 3′-rgn_cen vector after digestion with *Nhe*I and *Sal*I.

For DiCre recombination activity, the reporter parasites were cultured in 100 nM rapamycin-containing medium for 3 days. Genotyping assay was performed by PCR using MalP4205/1723 for GGe or MalP905/4202 for GGm to confirm deletion of sequence between *loxP* sequences.

### 2.5 Fluorescence microscopy

GG, CH, GH and CG reporter parasites were synchronously cultured for 3 days after 5% sorbitol treatment, as previously described [25]. Schizont and trophozoite stage cells were purified by Percoll-sorbitol gradient centrifugation following the previously-reported procedures [25]. The collected cells were stained with 1 μg/mL Hoechst 33342-containing PBS for 1–2 min. Thin smear of the stained cells on glass plate was observed under the fluorescence microscope BX60 equipped with CCD camera DP73 (Olympus). 700–1400 cells for each strain were counted by two individuals. In the same exposure time, cells which show saturated fluorescence signal of mNG in the image were categorized into “bright”, while those of no signals or reddish autofluorescence were categorized into “negative”. The other mNG signal-positive cells were categorized into “dim”. The signal intensity of each cell was measured using the image processing software Fiji (https://imagej.net/software/fiji/), in GH and CH cell lines.

### 2.6 Chromatin immunoprecipitation (ChIP) assays

Each of the reporter strains was synchronously cultured for 3 days after 5% sorbitol treatment. 3–10 × 10^8^ parasites were fixed with 1% formaldehyde at 30 °C for 1 h. The fixed cells were incubated in 0.83% NH_4_Cl solution on ice to remove RBC components. The naked parasites were lysed in the buffer containing 50 mM Tris-HCl (pH 8.0), 1% SDS and 0.5 mM EDTA (pH 8.0), and applied to sonication using S220 Focused-ultrasonicator (Covaris), to shear the genomic DNA. The chromatin fragments were incubated with anti-H3K9me3 antibodies (Active Motif) conjugated with magnetic beads (Thermo Fisher Scientific) at 4 °C overnight to precipitate H3K9me3-marked nucleosomes. DNA fragments were recovered from the nucleosomes after RNase and proteinase K treatment.

Twenty-five pg of the ChIP DNA fragments were applied to quantitative PCR (qPCR) using MalP4219/4223, MalP4233/4234, MalP1291/1292, MalP1295/1296, MalP1297/1298, MalP1865/1866 and MalP4928/4929 to amplify *mNG*, *hDHFR*, *serine-tRNA ligase* (*STL;* PF3D7_0717700), *fructose-bisphosphate aldolase* (*FBA*; PF3D7_1444800), *actin* (*ACT*; PF3D7_1246200), *var46* (PF3D7_1150400) and a backbone sequence of pRep_HSPpro-hDHFR_cen vector, respectively. The qPCR was performed using PowerSYBR Green PCR Master Mix (Thermo Fisher Scientific) and QuantStudio 3 real time PCR system (Thermo Fisher Scientific).

ChIP seq library production was performed using the recovered DNA fragments as follows. Two ng of ChIP DNA fragments were applied to size selection by SPRI magnetic beads (Beckman Coulter). The selected fragments (∼300 bp) were end repaired, adapter-ligated and amplified using KAPA HyperPrep Kit (Kapa Biosystems), following manufacturer's instruction.

### 2.7 Sequencing and data analysis of ChIP seq libraries

The libraries were converted to ones optimized for DNBSEQ, using MGIEasy Universal Library Conversion Kit (App-A). The resultant libraries were sequenced with 2 × 100 bp paired-end mode on DNBSEQ-G400RS platform (MGI, Shenzhen, China). Raw fastq files were aligned with Bowtie2 (v 2.4.2), onto the reference genome sequence of *P. falciparum* 3D7 (PlasmoDB 46), which is combined with each of the artificial chromosome sequences. Peaks for all the ChIP seq data were called using MACS2 (v 2.2.7.1) under a P-value cutoff of 1e–05. The obtained bedgraph files were all visualized with the Integrative Genome Viewer (IGV v 2.4.11). ChIP seq data were deposited on DNA Data Bank of Japan (DDBJ) (www.ddbj.nig.ac.jp/) under accession nos. DRR446188-DRR446199.

## 3 Results and discussion

### 3.1 Assessment of reporter silencing in artificial chromosomes

To assess heterochromatin formation on artificial chromosomes without any effects from the endogenous *AP2-G* locus, we first tried to remove the entire heterochromatic region of the *AP-2G* locus using the CRISPR/Cas9 system (Fig. 1A, S1). Co-introduction of an sgRNA-expressing vector and donor DNA fragment targeting upstream- and downstream sites of the heterochromatic region removed 14.3 kb of sequence, including 4.4 kb upstream- and 2.6 kb downstream of the *AP2-G* coding sequence (CDS) (Fig. S1A). The successful sequence deletion was confirmed by diagnostic PCR (Fig. S1B), and the resultant parasite was designated AP2-Gdel.

We next prepared reporter constructs, which contain an expression cassette of the fluorescence protein mNeonGreen (mNG), to investigate whether the 5′- or 3′-flanking sequences of the *AP2-G* CDS contributes to epigenetic gene silencing (Fig. 1A). In construct GG, 4.9 kb upstream- and 1.9 kb downstream flanking regions of the *AP2-G* CDS are used as a promoter (*AP2-G 5′-rgn*) and terminator (*AP2-G 3′-rgn*) respectively. The control constructs, in which either one or both of *AP2-G 5′-rgn* and *AP2-G 3′-rgn* are swapped with *calmodulin* gene promoter (*Cam pro*) and *P. berghei heat shock protein* gene terminator (*PbHSP ter*) respectively, were generated as shown in Fig. 1A (constructs GH, CG and CH). Each construct was introduced into the AP2-Gdel strain as an artificial chromosome.

**Fig. 1:**
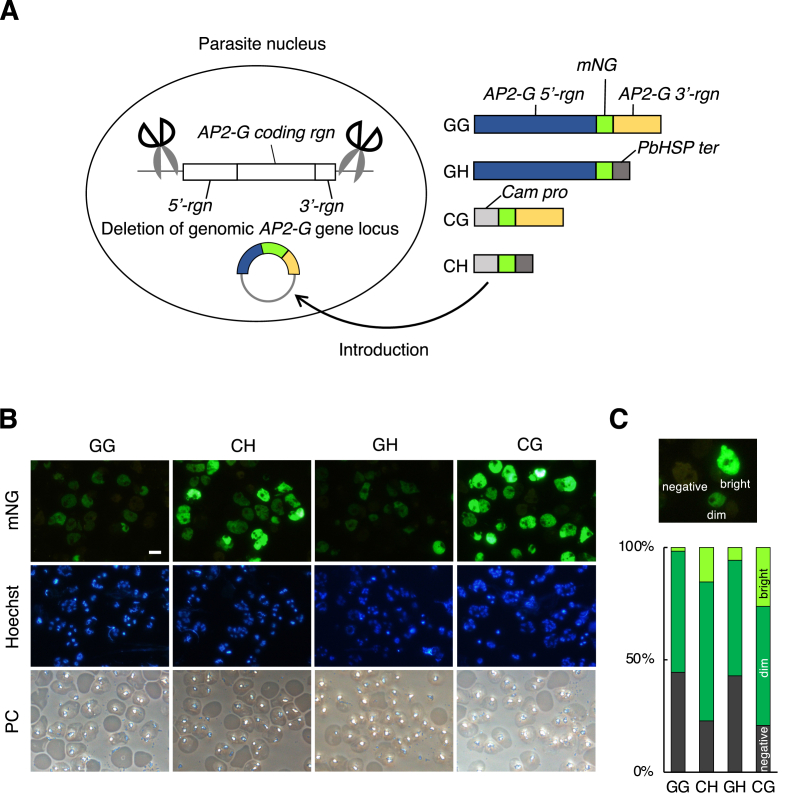
Development of reporter system using artificial chromosome (**A**) Schematic of reporter line production. Each artificial chromosome vector containing GG, GH, CG or CH construct was introduced into the *AP2-G* deleted parasite. (**B**) Fluorescence microscopy of reporter strains. Schizont stage parasites were collected and Hoechst stained. For each reporter strain, identical-field images of mNG signal, Hoechst staining and phase contrast (PC) were captured. The scale bar represents 10 μm. (**C**) The reporter parasites were counted for each of signal intensities categorized into “bright”, “dim” and “negative".

When observed at the schizont stage, CH and CG parasites displayed strong mNG fluorescence, whereas most GG and GH parasites displayed only a weak mNG signal (Fig. 1B). We next categorized the signal intensity of each parasite into “negative”, “dim” or “bright” as shown in Fig. 1B (see Materials and methods). As a result, the total scores of “negative” and “dim” in GG and GH were greater than those of CH and CG (Fig. 1B).

### 3.2 Detection of H3K9 trimethylation in the artificial chromosomes

We reasoned that the comparatively large number of “negative” and “dim” cells present in the cloned GG and GH cell lines may be due to epigenetic silencing of *mNG* in them rather than to differences in the strength of promoter activity between *AP2-G 5′-rgn* and *Cam pro*, because the signal intensity of their “bright” cells was comparable with each other (Fig. S2). To validate the hypothesis, a chromatin immunoprecipitation (ChIP) assay using anti-H3K9me3 antibodies and quantitative PCR (qPCR) using primers for the genes on the artificial chromosome (*mNG* and human *dihydrofolate reductase* (*hDHFR*)) was performed. A heterochromatinized gene (*var46*) and actively-expressed genes (*serine-tRNA ligase* (*STL*), *fructose-bis-phosphate aldolase* (*FBA*) and *actin* (*ACT*)) on the native chromosomes were used as positive and negative controls respectively (Fig. S3). GG and GH lines showed preferential amplification of *mNG* and *hDHFR* (Fig. 2A). This trend was reconfirmed in another GG cell line that was produced independently of Fig. 2A (Fig. S3B). In the CH and CG cell lines, however, *mNG* and *hDHFR* amplification were comparable to those of the negative controls (Fig. 2A). This shows two facts as follows. First, *de novo* H3K9 trimethylation occurred on GG and GH construct vectors, although they were artificial chromosomes introduced as naked DNA. Second, the sequence of *AP2-G 5′-rgn*, but not *3′-rgn*, contributes to the H3K9 methylation, leading to the mNG silencing as shown in Fig. 1B.

**Fig. 2:**
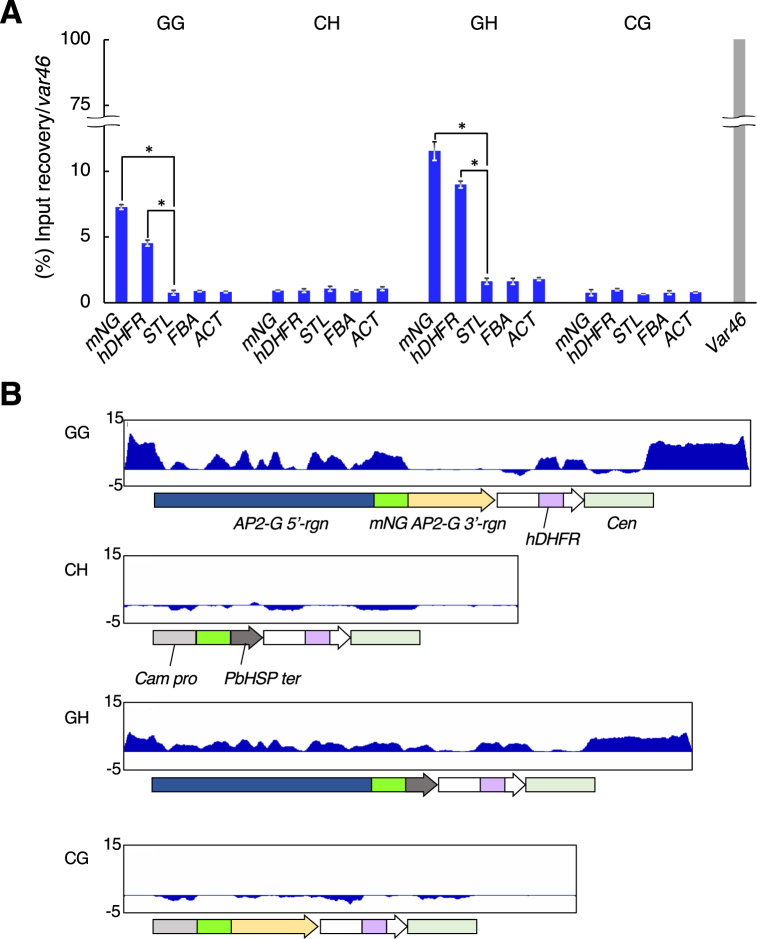
H3K9 methylation on the introduced artificial chromosomes (**A**) ChIP-qPCR assays for H3K9me3 on GG, CH, GH and CG strains. The result is shown as a percentage of recovery normalized against *var46* (100%) of each graph set. Each bar shows the average of three technical replicates. The error bars represent S.D. * Student's T-test, p < 0.05. (**B**) ChIP seq analyses on the artificial chromosome of reporter strains. Landmark constructs are shown under each bedgraph.

Supporting this observation, ChIP sequencing (ChIP-seq) of the same samples as qPCR showed peaks for H3K9me3 in most regions of the GG and GH vectors, while no obvious peaks were detected in those of CH and CG (Fig. 2B). Because the peaks were distributed not only in the construct regions but also in those of vector backbone, sequence-independent spreading of H3K9 methylation throughout the vector may have occurred in GG and GH, like in fission yeast [26]. However, the artificial chromosome's active promoter from the *hDHFR* expression cassette (*HSP70 pro*), as well as the centromere (*Cen*) and *AP2-G 3′-rgn* (which are H3K9me3-free sequences in the native chromosomes) were H3K9me3-negative, which may suggest that eviction of H3K9me3 occurred at these loci following the H3K9me3 spreading. This implies that epigenetic contexts of GG and GH vectors mostly reflect those of native chromosomes.

### 3.3 Identification of the sequence element inducing H3K9me3 deposition in *AP2-G* promoter

Since *AP2-G 5′-rgn* was found to be critical for *de novo* H3K9 methylation, we next tried to identify a specific sequence element that plays an important role in *AP2-G* silencing. To this end, a series of reporter strains possessing a modified GG construct (Del_I-VI), in which *AP2-G 5′-rgn* sequence is partially deleted, was prepared (Fig. 3A). When ChIP-qPCR assays were performed as described above, Del_II and III strains showed only weak amplification of *mNG* and *hDHFR*, while those of the other strains were comparable with GG and GH (Fig. 3B). A comparison of the results among Del_I-IV suggested that 5′- and 3′-ends of silencer sequence should be within the 3.3–4.4 kb and 0.1–2.4 kb upstream regions of transcription start site (TSS) respectively. The Del_V strain, in which the putative silencer-containing sequence is within a 1.1–3.8 kb region from TSS, showed obvious H3K9 methylation (Fig. 3A–B). The Del_VI strain, in which the putative silencer-containing sequence is further shortened, also maintained the silencing activity, suggesting this ∼2.0 kb sequence corresponds to the minimal silencer element (Fig. 3A–B).

**Fig. 3:**
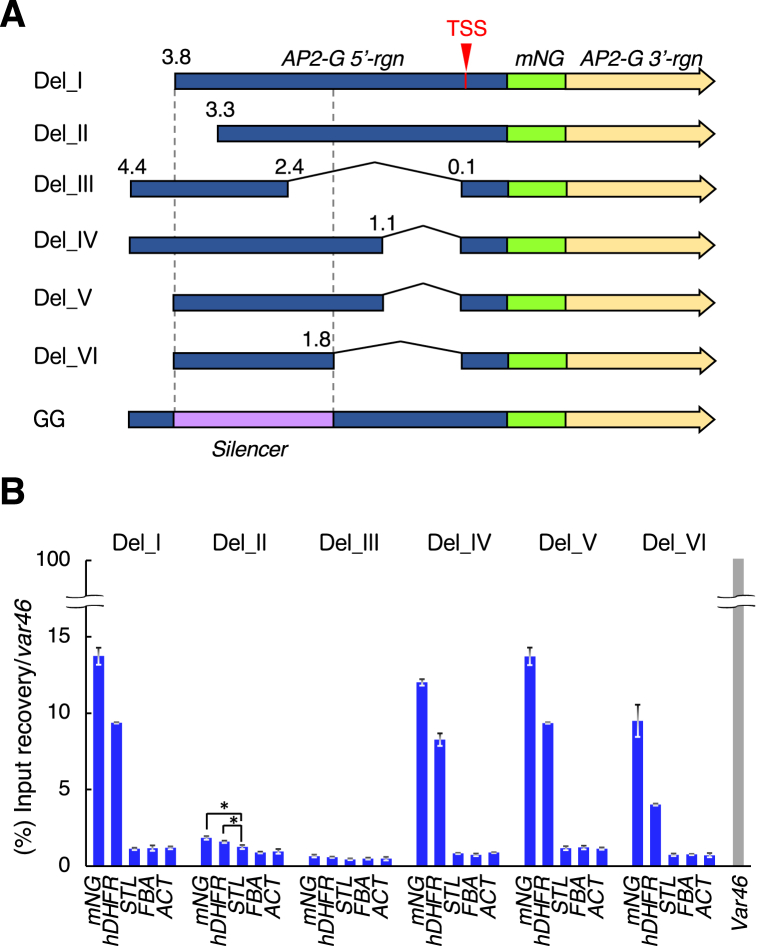
Identification of silencer sequence in *AP2-G* 5′ region (**A**) Schematics of the partially-deleted GG constructs Del_I-VI. The numerals indicate distance (kb) from transcription start site (TSS). (**B**) ChIP-qPCR assays for H3K9me3 on Del_I-VI strains. The result is shown as a percentage of recovery normalized against *var46* (100%) of each graph set. Each bar shows the average of three technical replicates. The error bars represent S.D. * Student's T-test, p < 0.05.

### 3.4 Functional dissection of the silencer sequence

When the ChIP-qPCR results were compared between Del_II and III, we noted slightly-stronger amplification of *mNG* and *hDHFR* in Del_II, than those of Del_III (Fig. 3B, asterisks). In support of this, a ChIP-seq analysis showed sparse peaks for H3K9me3 in the vector backbone, *mNG* and *hDHFR* sequences of Del_II, while Del_III showed similar peaks in a restricted region of the vector (Fig. S4A, peaks underlined in red). The notable H3K9me3 deposition in the Del_II vector was also confirmed by ChIP-qPCR assay (Fig. S4B). Those results above may suggest that although the Del_II vector experienced *de novo* H3K9 methylation, the H3K9me3 was destabilized due to the sequence deletion in the *AP2-G 5′-rgn*. This prompted us to hypothesize that the deleted sequence of *AP2-G 5′-rgn* in Del_II and III participates in stability and *de novo* deposition of H3K9me3 respectively. To validate this hypothesis, we performed conditional sequence deletion assays based on rapamycin-inducible Cre/loxP system, in which FKBP12 and FRB proteins are dimerized into a functional Cre recombinase that deletes DNA sequence between loxP sites, in the presence of rapamycin [19]. As shown in Fig. 4A, we generated two new reporter strains designated GGe and GGm, in which partial *AP2-G 5′-rgn* sequences, shown to be essential for H3K9 methylation in Del_III and II respectively, reside between loxP sites. When ChIP-qPCR assays were performed after culturing in rapamycin-free medium, both strains showed obvious amplification of *mNG* and *hDHFR* genes, comparable with those of GG. This indicates that none of the loxP sequences inserted in *AP2-G 5′-rgn* affected H3K9 methylation itself in both strains. After culturing in rapamycin containing medium, we succeeded in partial deletion of *AP2-G 5′-rgn* in both strains, and performed ChIP-qPCR assays for them similarly (Fig. 4B). Unexpectedly, no significant changes were detected in H3K9 methylation level between before and after rapamycin treatment for approximately 3 days (66 and 72 h in GGe and GGm respectively) (Fig. S5). We attributed this result to insufficient duration after sequence deletion and mixed populations of parasites that succeeded or failed in sequence deletion. To precisely evaluate H3K9 methylation level before and after sequence deletion, we cloned the parasites after rapamycin treatment into sequence deletion-positive (Del (+)) and negative (Del (−)) populations, for each of GGe and GGm strains, and performed ChIP-qPCR assays. The Del (+) GGe parasites maintained H3K9me3 at a detectable level, while those of GGm showed significant decrease of H3K9me3 (Fig. 4B). This strongly suggests that the 5′-end site of the silencer is required for maintenance of H3K9me3, while the 3′-end participates in initiating H3K9 methylation but is dispensable for its maintenance.

**Fig. 4:**
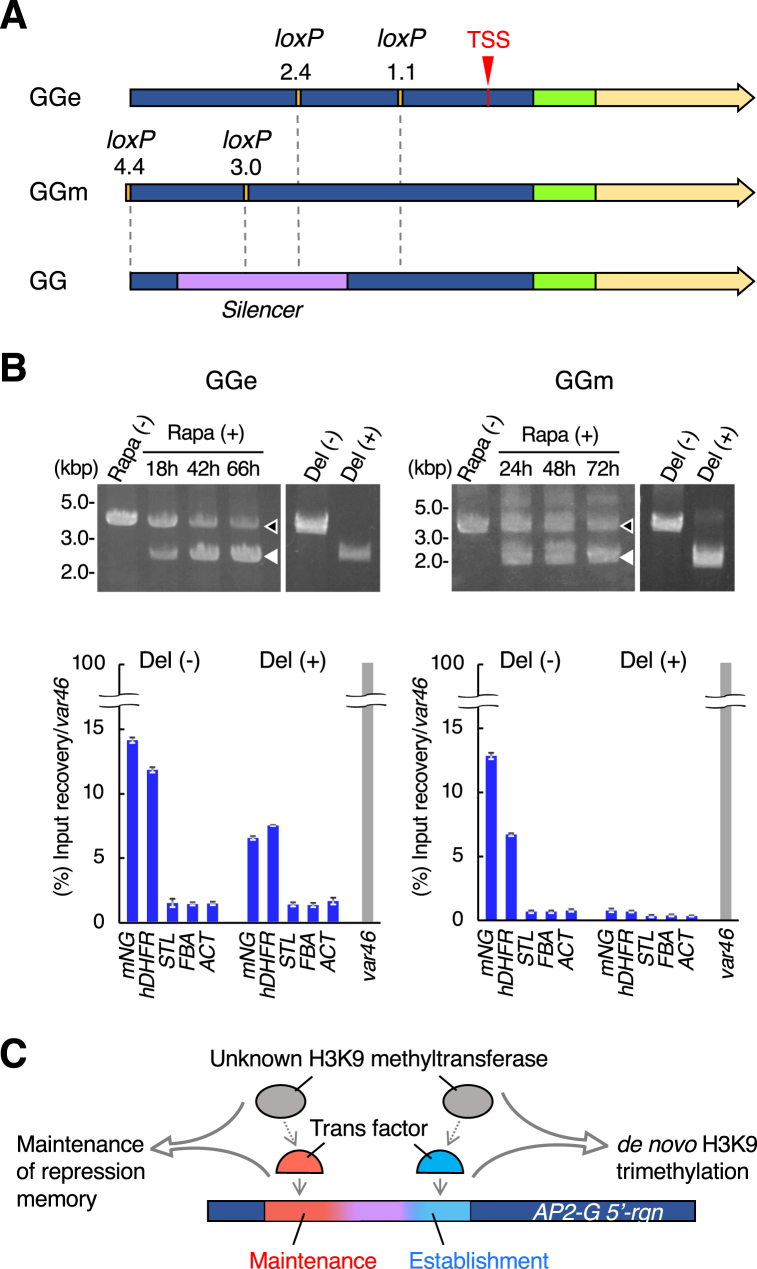
Functional dissection of the silencer sequence (**A**) Schematics of loxP-site-inserted GG constructs. The numerals indicate distance (kb) from transcription start site (TSS). (**B**) (Top) Diagnostic PCRs showed amplified products before (black arrowheads) and after (white arrowheads) sequence deletion by rapamycin (Rapa) treatment for 18 h, 42 h and 66 h (GGe) or 24 h, 48 h and 72 h (GGm). (Bottom) ChIP-qPCR assays on deleteion-negative (Del (−)) and positive (Del (+)) parasites cloned from each of Rapa-treated GGe and GGm strains. The result is shown as a percentage of recovery normalized against *var46* (100%) of each graph set. Each bar shows the average of three technical replicates. The error bars represent S.D. Digitally-captured images with DNA ladder marker are shown in Supplementary files “GGetimecourse”, “GGeGGmtimecourse”, “GGecloned” and “GGmcloned”. (**C**) Model of the silencer functions. 5′- and 3′-end regions of the silencer may function as a *cis*-regulatory elements to control establishment and maintenance of heterochromatin in *AP2-G* locus, respectively. Each region is recognized by a specific *trans* factor, which recruits H3K9 methyltransferase.

## 4 Conclusions

Malaria parasites lack the non-homologous end-joining pathway, so genome editing in the parasite requires the use of homologous recombination of donor DNA fragments [27]. Heterochromatin is known to be a structure that participates in inhibition of aberrant homologous recombination [28], and therefore, direct dissection of heterochromatinized sequences has been technically difficult in *Plasmodium* species. In this study, we established a strategy to assess *de novo* deposition of H3K9me3-marked nucleosomes on an artificial chromosome vector introduced into *P. falciparum* as naked DNA. ChIP-qPCR assays sensitively detected H3K9me3 on GG and GH vectors in the absence of the endogenous *AP2-G* locus, demonstrating the utility of artificial chromosome for assessment of *P. falciparum* heterochromatin formation (Fig. 1). In fission yeast, a similar artificial “mini-chromosome” has contributed to understanding how heterochromatin is established, spread, and maintained in the centromeric region [2]. We expect that our method will serve as a versatile tool to elucidate epigenetic gene regulation in malaria parasites in future.

In this study, we succeeded in identifying a specific DNA sequence that acts as a silencer of *P. falciparum AP2-G* that is critical to both *de novo* H3K9 methylation and its maintenance. The next step will be to identify the silencer-binding protein factors that recruit an unknown H3K9 methyltransferase, to further elucidate the mechanism of heterochromatin formation in the *AP2-G* promoter (Fig. 4C). In fruit flies, the silencer element PRE participates in PRC2 recruitment to methylate H3K27, but not H3K9, and is required for maintenance of silencing as well [10,11]. No silencer element has been identified in *Tetrahymena*, instead, the PRC1/2-like EZL1 complex with both H3K9 and H3K27 methylation activity has been shown to be recruited to target regions via an RNAi pathway, similar to H3K9 methylation in fission yeast [29,30]. Malaria parasites do not have an RNAi pathway and no PRC-like complexes have been reported in the parasite to date. It seems that the H3K9me-inducing silencer element identified in this study depends on a yet-unknown molecular system, distinct from that of the fruit fly and *Tetrahymena*. Recently, Moazed and colleagues demonstrated that the maintenance of silencing memory in fission yeast, established by H3K9 methylation, is dependent on a set of specific DNA sequence elements termed “maintainer” [7]. The silencer element in *AP2-G* identified in this study also appears to act as a maintainer. Interestingly, the maintainer in fission yeast also shows weak activity as a silencer to induce *de novo* H3K9 methylation when the anti-silencing factor Epe1 is knocked-out [7]. It is well-established that H3K9 methylation-mediated silencing forms the core mechanism for transcription regulation in multicellular organisms [[31], [32], [33]]. Kruppel-associated box domain zinc finger proteins (KRAB-ZFPs) are one of the well-known transcription regulators that represses transposable element derived sequences, contributing to cell reprogramming and differentiation [31,34,35]. As AP2-G plays its own key role in cell differentiation (i.e. asexual-to-sexual conversion) of malaria parasites, the aforementioned reports have inspired us to understand its sequence-dependent silencing system. Although there is still much to be learned about the heterochromatinization process in malaria parasites, the apparent simplicity of the silencing system, which involves no RNAi or DNA methylation, makes this organism an important model for understanding the diverse mechanisms that eukaryotes have evolved for gene regulation using H3K9me-marked heterochromatin.

## Funding sources

This work was supported by 10.13039/501100001691JSPS KAKENHI grant nos. JP21K06987 (T.M.) and JP22K15449 (M.N.), and 10.13039/501100001700MEXT LEADER grant no. JPMXS0320200151 (M.N.).

## Inclusion and diversity

We support inclusive, diverse, and equitable conduct of research.

## Author contribution statement

Toshiyuki Mori: Conceived and designed the experiments; Performed the experiments; Analyzed and interpreted the data; Contributed reagents, materials, analysis tools or data; Wrote the paper. Mai Nakashima: Performed the experiments; Contributed reagents, materials, analysis tools or data; Wrote the paper.

## Data availability statement

Data associated with this study has been deposited at ChIP seq data were deposited on DNA Data Bank of Japan (DDBJ) (www.ddbj.nig.ac.jp/) under accession nos. DRR446188-DRR446199.

## Declaration of competing interest

The authors declare that they have no known competing financial interests or personal relationships that could have appeared to influence the work reported in this paper.

## Figures and Tables

**figs1:**
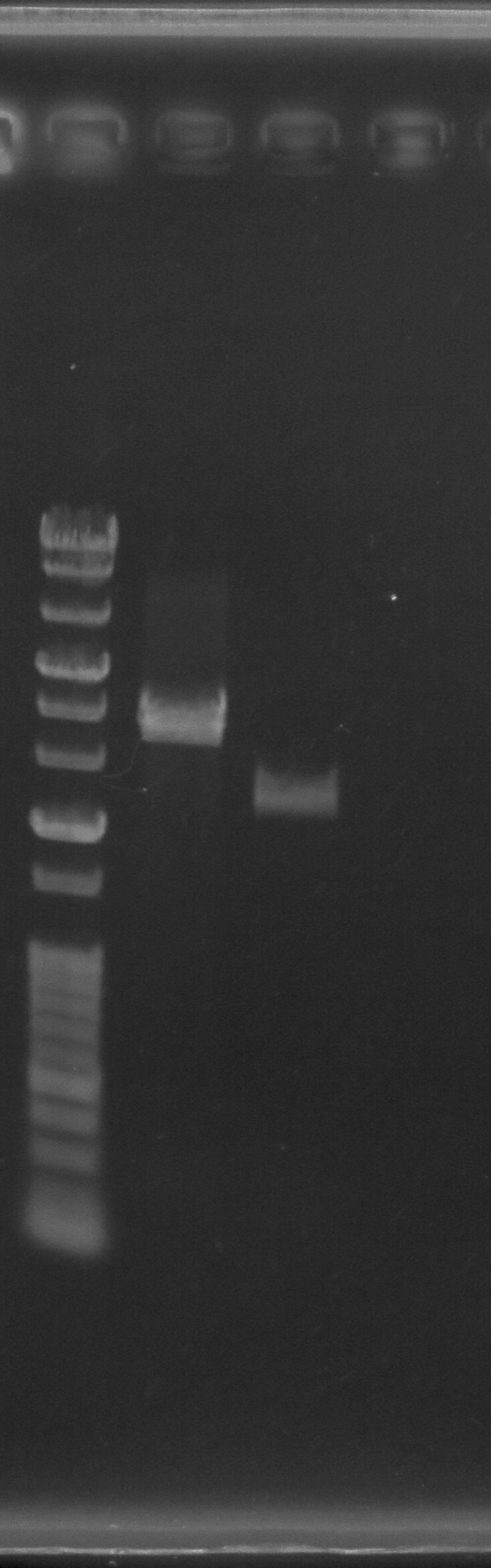
GGecloned.

**figs2:**
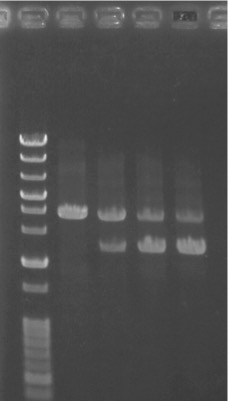
GGetimecourse.

**figs3:**
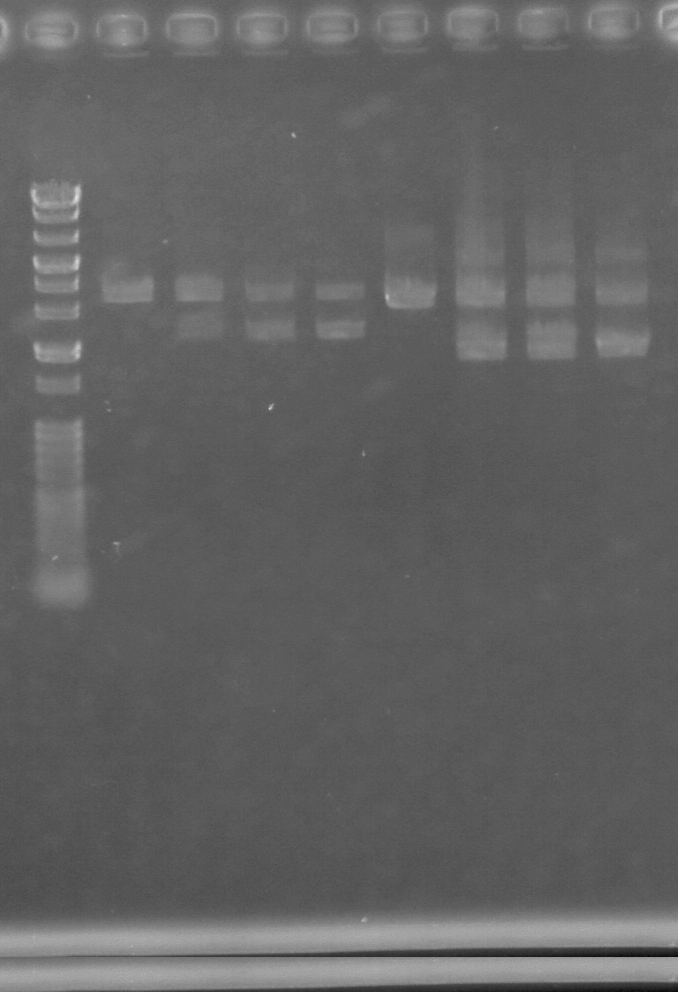
GGeGGmtimecourse.

**figs4:**
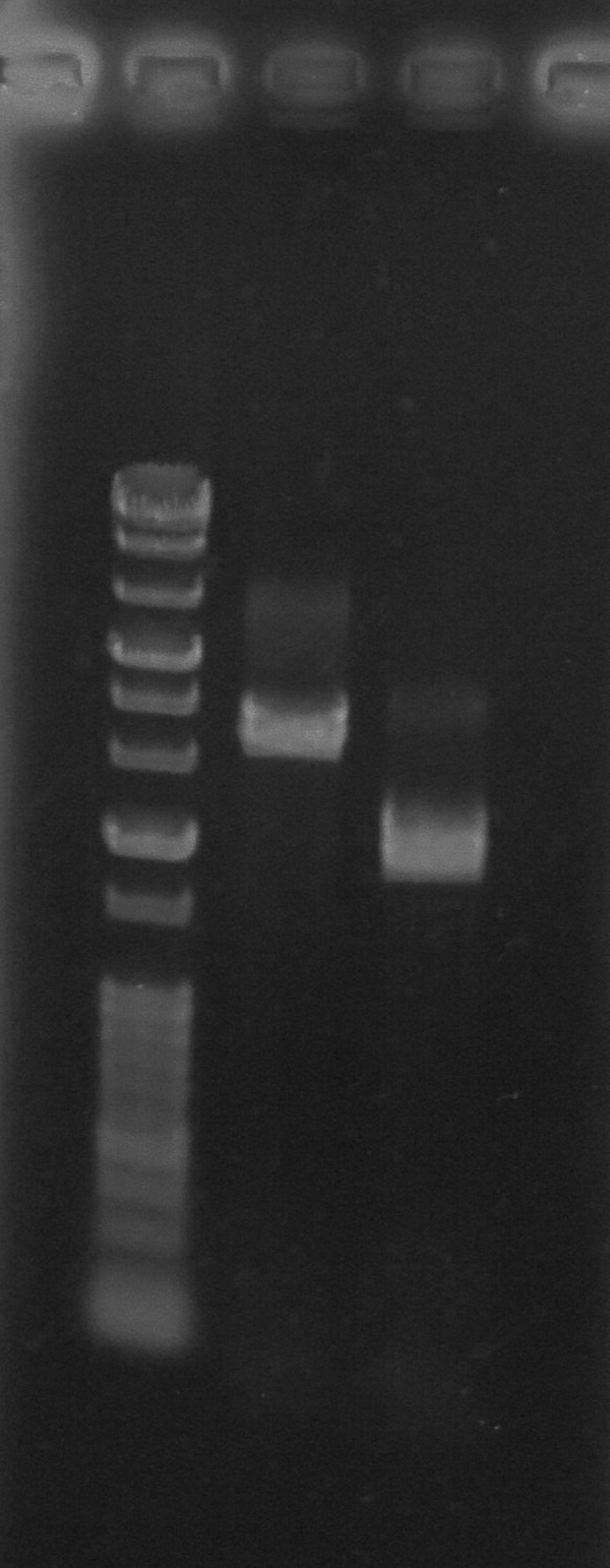
GGmcloned.

**figs5:**
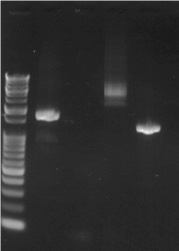
AP2Gdel.
