# Supplementary material for: Sequence-dependent heterochromatin formation in the human malaria parasite Plasmodium falciparum
Source: Heliyon. 2023 Aug 16;9(9):e19164. doi: 10.1016/j.heliyon.2023.e19164 (PMC10480601; doi:10.1016/j.heliyon.2023.e19164)
Supplement: Multimedia component 1 [file mmc1.pdf]

A

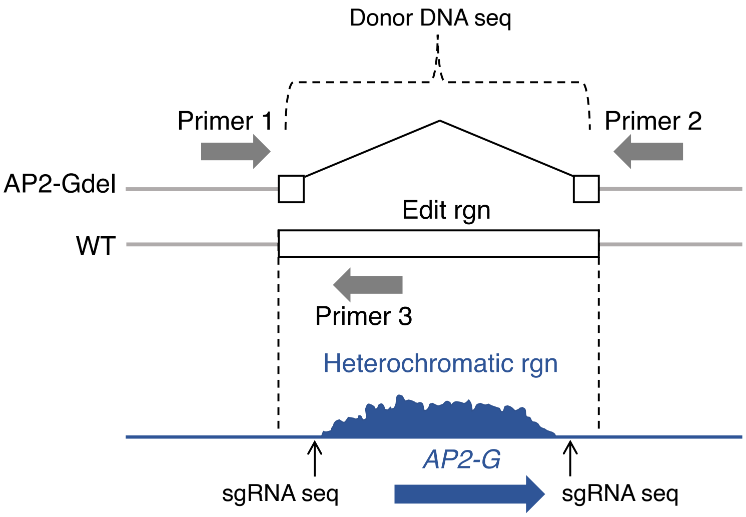

B

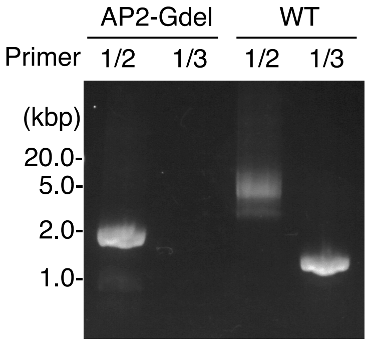

Fig. S1. Deletion of *AP2-G* locus by CRISPR/Cas9 system
(**A**) Schematic of genome-editing procedures. The gray arrows represent position and direction of primers used for diagnostic PCR shown in (**B**). (**B**) Diagnostic PCR on *AP2-G* deleted (AP2-Gdel) parasites confirmed successful deletion of the entire *AP2-G* locus. In the left side lane of wild type (WT) parasite, incomplete PCR products were detected due to extremely-long distance between primer-annealing sites. Digitally-captured image with DNA ladder marker is shown in Supplementary file "AP2Gdel".

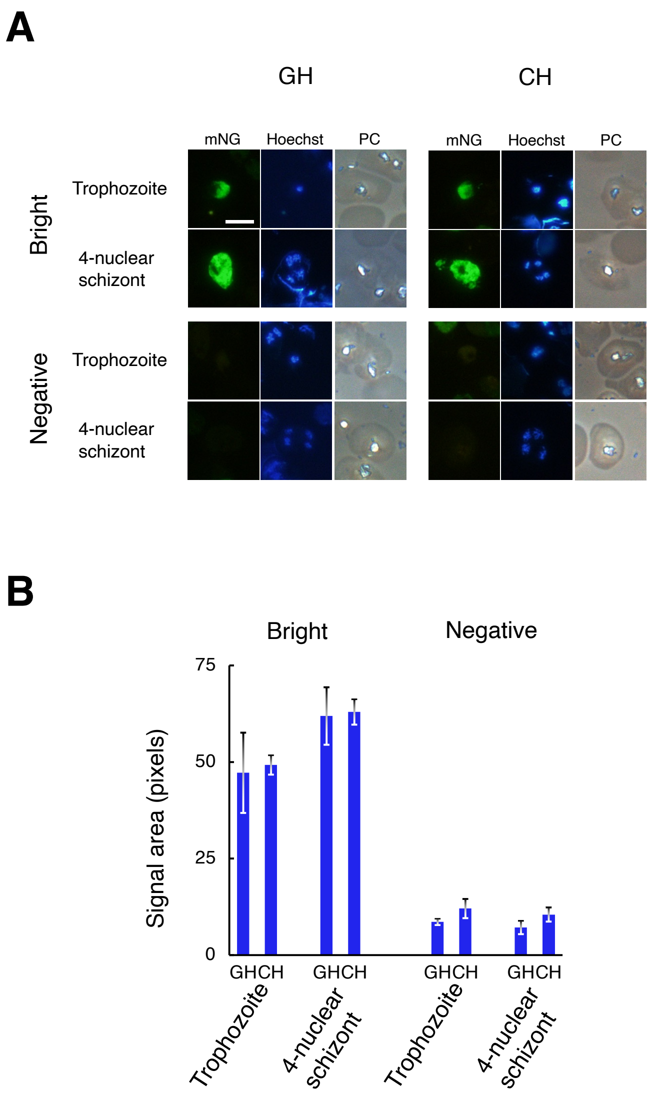

Fig. S2. Validation of mNG signals defined as "bright" and "negative"
(**A**) Fluorescence images of trophozoites and 4-nuclear schizonts showing "bright" mNG signal or no positive signals, in GH and CH cell lines. The scale bar represents 5 µm. (**B**) Comparison of fluorescence intensity in cells categorized into "bright" or "negative" between GH and CH cell lines, using the image processing software Fiji. Each bar shows the average on three different cell images captured in the same exposure time. The error bars represent S.D.

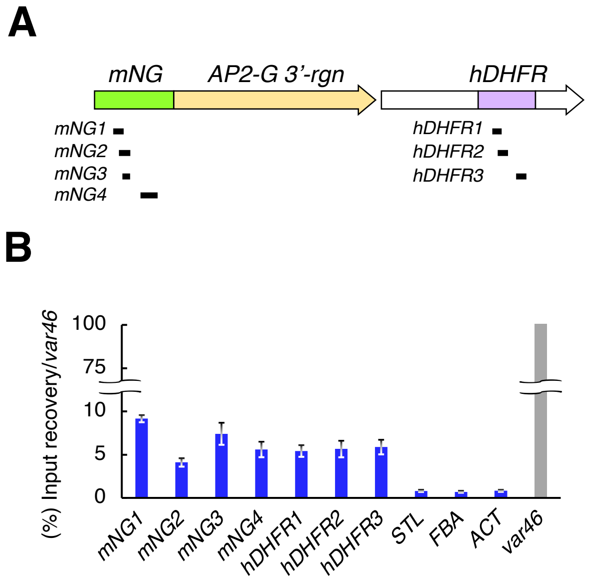

Fig. S3. Comparison of H3K9me3 abundance between native- and artificial chromosome genes
(**A**) Schematic of partial region of the GG vector. The regions amplified in ChIP qPCR assays in (**B**) are represented with black bars. The regions *mNG1* and *hDHFR1* were used for ChIP qPCR assays shown in Fig 2, 3, 4, S4 and S5. (**B**) ChIP qPCR assay for H3K9me3 on GG strain. The result is shown as a percentage of recovery normalized against *var46* (100%). Each bar shows the average of three technical replicates. The error bars represent S.D.

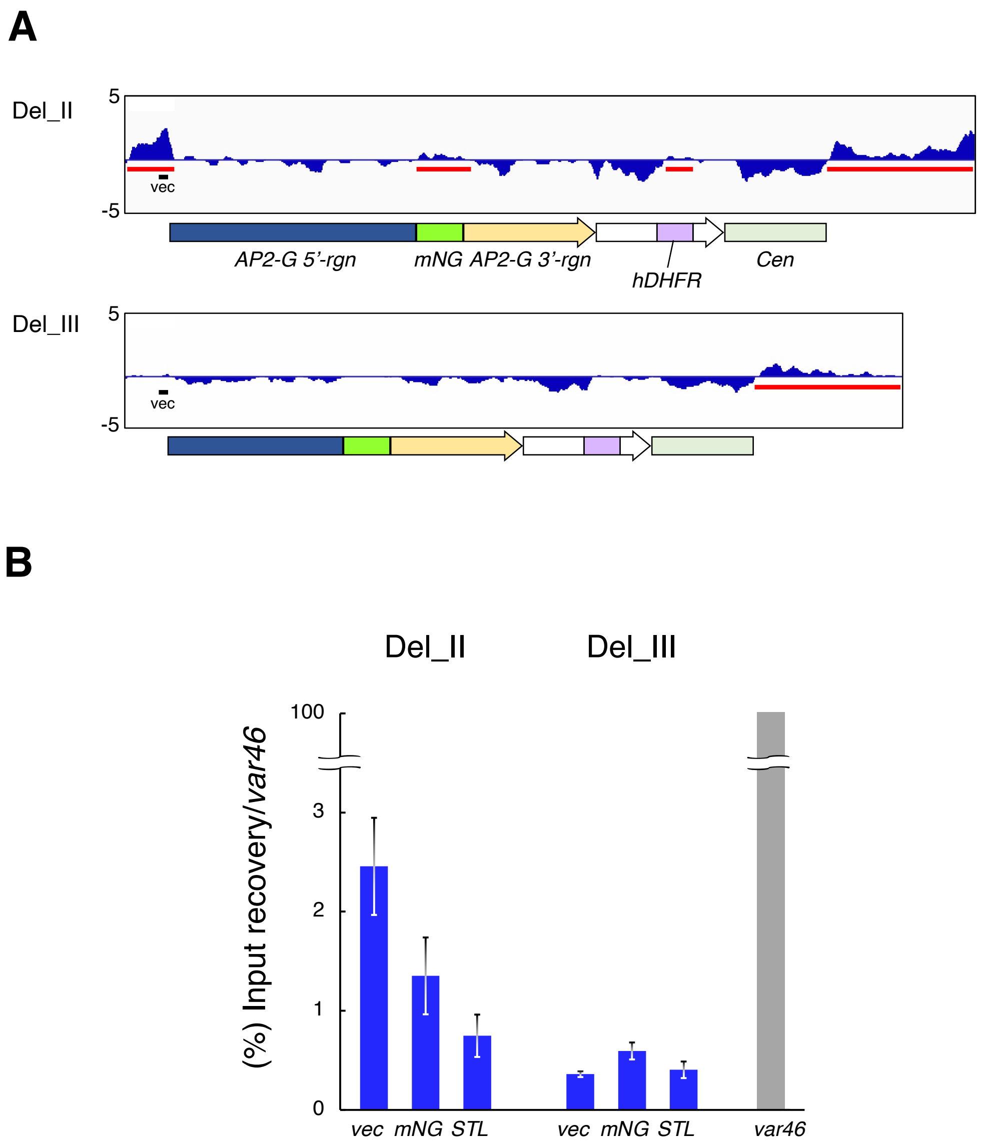

Fig. S4. H3K9me3 detected in Del_II chromosome vector
(**A**) ChIP seq analyses of Del_II and III construct vectors. The regions showing obvious peaks are underlined in red. The regions underlined in black (vec) were amplified in ChIP qPCR assay shown in (**B**). (**B**) ChIP qPCR assay for H3K9me3 on Del_II and III strains. The result is shown as a percentage of recovery normalized against *var46* (100%) of each graph set. Each bar shows the average of three technical replicates. The error bars represent S.D.

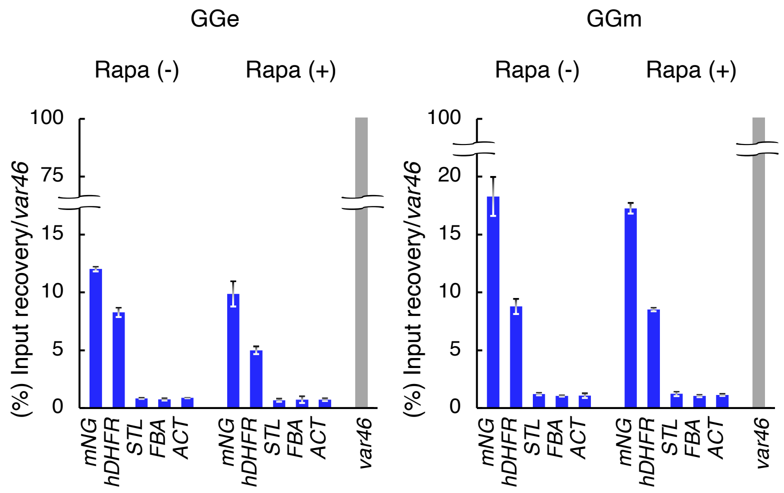

Fig. S5. Comparison of H3K9me3 levels between before and after rapamycin treatment on GGe and GGm strains

Parasites before (Rapa (-)) and after (Rapa (+)) rapamycin treatment for approximately 3 days (66 h and 72 h in GGe and GGm respectively) were applied to ChIP qPCR assays. The result is shown as a percentage of recovery normalized against *var46* (100%) of each graph set. Each bar shows the average of three technical replicates. The error bars represent S.D.

Oligo name	Sequence	Experiments
MalP905	CCAGTCACGACGTTGTAAAACGACGGCCAG	Production of reporter constructs
MalP1291	AAGTAGCAGGTCATCGTGGTT	ChIP assays
MalP1292	TTCGGCACATTCTTCCATAA	ChIP assays
MalP1295	TGTACCACCAGCCTTACCAG	ChIP assays
MalP1296	TTCCTTGCCATGTGTTCAAT	ChIP assays
MalP1297	AGCAGCAGGAATCCACACA	ChIP assays
MalP1298	TGATGGTGCAAGGGTTGTAA	ChIP assays
MalP1715	CTTATTTATACAATTCATCCATACCCATAACATCAGTAAATG	Production of reporter constructs
MalP1717	TTTATTTATACAATTTTTATTTATACAATTCATCC	Production of reporter constructs
MalP1723	TAACTGGACCATCTGCTGGAAAACC	Production of reporter constructs
MalP1865	TGCTGAAGACCAAATTGAGC	ChIP assays
MalP1866	TTGTTGTGGTGGTTGTTGTG	ChIP assays
MalP1970	TATTGCATTTCGGTTTGGGTCATT	CRISPR/Cas9 genome editing
MalP1971	AAACAATGACCCAAACCGAAATGC	CRISPR/Cas9 genome editing
MalP2073	GACGAGCTGTACAAGTGAACTAATAAAAAAAAAAAACATTAAACAGGAC	CRISPR/Cas9 genome editing
MalP2278	CGGGCCCCCCCTCGAGCGTGAGTTTTTTCTTTTCCCATATGTTTTATAAAATTTTTATTTATTTATAAGC	CRISPR/Cas9 genome editing
MalP2279	CGATGCAGTTTAGCGAACCAACCATGGTTTTATAAAATTTTTATTTATTTATAAGCAA	CRISPR/Cas9 genome editing
MalP2622	CGGGCTGCAGGAATTGTTGAGGCCTTATTCCAGGAATACC	CRISPR/Cas9 genome editing
MalP2704	CGGGCCCCCCCTCGAGGCGTATACGTCTTATGCACAAATATAGT	CRISPR/Cas9 genome editing
MalP3068	TATAGGGCGAATTGGGTACCGG	Production of reporter constructs
MalP3070	GTTGGTTCGCTAAACTGCATCG	Production of reporter constructs
MalP3071	CGTTACTAGTGGATCCGAAATTGAAGG	Production of reporter constructs
MalP3091	TATTTATGCGCTCGAGTCGACAAAATACCAATAATACCGTTTGG	Production of reporter constructs
MalP3247	ATTTTATAAAACATATGCTTTCAACTGAATGGTCCCC	CRISPR/Cas9 genome editing
MalP3248	TTTTATAAAACCATGGTGACCTTGTCCATTACCTTGATTG	CRISPR/Cas9 genome editing
MalP3249	TTTTATAAAACCATGGCCCCTAAGAAGAAGAGAAAGG	CRISPR/Cas9 genome editing
MalP3250	CTTGTACAGCTCGTCTCAGTTCAGCTTGCACCAGGC	CRISPR/Cas9 genome editing
MalP3251	GACAAGGTCACCATGGCCTAATAATACATATATTTTTAAAAACATTTTGTATGG	CRISPR/Cas9 genome editing
MalP3252	ATTTTATAAAACATATGATGGCCCCTAAGAAGAAGAGAAAGG	CRISPR/Cas9 genome editing

MalP3253	TATTATATAAATCCTTCAGTCCCCATCCTCGAGCAG	CRISPR/Cas9 genome editing
MalP3254	AGGATTTATATAATATATTTATGTACTCACAATGGGGTC	CRISPR/Cas9 genome editing
MalP3255	TCAGTTGAAAGCATATGATGAATACACATAAGTTGTTAATATATTTTATATGC	CRISPR/Cas9 genome editing
MalP3840	TATTGAAATAATTCTCAGAAGAAA	CRISPR/Cas9 genome editing
MalP3841	AAACTTTCTTCTGAGAATTATTTC	CRISPR/Cas9 genome editing
MalP4031	TATTGAAAAAGGAAAATTAAACAT	CRISPR/Cas9 genome editing
MalP4032	AAACATGTTTAATTTTCCTTTTTC	CRISPR/Cas9 genome editing
MalP4033	CTTGTTATTATATTTCTAACAATAGTAGCATG	CRISPR/Cas9 genome editing
MalP4034	TATTAATGATTAATGTATATTTAATATAGAACAATATTCTACTTTATC	CRISPR/Cas9 genome editing
MalP4035	CTATATTAAATATACATTAATCATTAATAGTATAATTAGCTTTATG	CRISPR/Cas9 genome editing
MalP4036	AATGAATAGGGAAGGTTAAAATGGTAACTG	CRISPR/Cas9 genome editing
MalP4039	CTCACTATAGGGCGAATTGGCCATGGGCTAGCGTTTCAAAAGGTGAAGAAGATAATATG	Production of reporter constructs
MalP4040	GTTATGGGTATGGATGAATTGTATAAATAAGTCGACTACCACAAAGGGGGAAACAACAGG	Production of reporter constructs
MalP4041	CCAGATATTTATGCGCTCGAGCCTCTTTTATGAAAAAATTGAAAACCAAG	Production of reporter constructs
MalP4051	CGTTTTCTTTTATTGAAATATATATTTTGGCATATAGG	CRISPR/Cas9 genome editing
MalP4052	GTATATATGTACGTAAATATATATATTTATAGGGT	CRISPR/Cas9 genome editing
MalP4059	TGAGCTAGCAAAACGCGTAAAGTCGACGTTTCAAAAGGTGAAGAAGATAATATG	Production of reporter constructs
MalP4060	TGACTCGAGTTATTTATACAATTCATCCATACCCATAACATCAGTAAATGC	Production of reporter constructs
MalP4115	GCTTATTTCTAATAAATATTAAAGATTATACATTCTATTAG	CRISPR/Cas9 genome editing
MalP4122	TGAGGCGCTAGCAAAACGCGTAAATAACAATAGTAGCATGCTCAATATATATTATGTCAAAG	Production of reporter constructs
MalP4123	CGGTGAGTCGACCTTAGCGGTCATCTTCTTAAATATTCC	Production of reporter constructs
MalP4202	GAGAAAATATAAACCTTATATATTAAAGATCATAAAACTAATTTTCCC	Conditional sequence deletion assay

MalP4205	GGGAAAATTAGTTTTATGATCTTTAATATATAAGG	Conditional sequence deletion assay
MalP4216	GGGTATGGATGAATTGTATAAATAAGATCTATTATTGTTCTGTACTTCTTTTGTG	Production of reporter constructs
MalP4219	TTTGCAATTTTCACCTTGGA	ChIP assays
MalP4223	GCAGCTTGAAATGGTGACAT	ChIP assays
MalP4227	CTATAGGGCGAATTGGCCATGGGCTAGCCAGGAAATATTATTTATAACAAGAG	Production of reporter constructs
MalP4228	CATATTATCTTCTTCACCTTTTGAAACGTCGACCTTAGCGGTCATCTTGTCTGCCATTGATATATTTCTATTAGG	Production of reporter constructs
MalP4233	CCAGAGAATGACCACAACCT	ChIP assays
MalP4234	GGAATGGAGAACCAGGTCTT	ChIP assays
MalP4499	CTTCTTCACCTTTTGAAACGTCGACCTTAGCGGTCATCTTCTTAAATATTCC	Production of reporter constructs
MalP4642	ATAGGGCGAATTGGCCATGGGCTAGCTAACAATAGTAGCATGCTCAATATATATTATGTCAAAG	Production of reporter constructs
MalP4643	GCACATAATATGTAAAATAGTTATATGGACAATGCACTTTTTGAGTACAG	Production of reporter constructs
MalP4646	ATAGGGCGAATTGGCCATGGGCTAGCGTGCACATATATTTTAATTAGCTAGACGTAC	Production of reporter constructs
MalP4710	GGGCGAATTGGCCATGGGCTAGCATAACTTCGTATAGCATACATTATACGAAGTTATTAACAATAGTAGCATGCTCAAT	Production of reporter constructs
MalP4721	ATAGGGCGAATTGGCCATGGGCTAGCTATGAAAAAGAAAAAAAAAAGGAAAATTAAACATTGGAGAATTG	Production of reporter constructs
MalP4726	GCACATAATATGTAAAATAGTTATATGGAAGTATTTATATCTCTCACGGTTCATATC	Production of reporter constructs
MalP4731	GATATGAACCGTGAGAGATATAAATACTTATAACTTCGTATAATGTATGCTATACGAAGTTATTTTTTTTTTTTTTTTTCATAAACAAAG	Production of reporter constructs
MalP4732	AAGTATTTATATCTCTCACGGTTCATATCTT	Production of reporter constructs
MalP4748	TTTTTTTTATTAAGCTTGTACAACTTTTAAATG	Production of reporter constructs
MalP4749	CATTTAAAAGTTGTACAAGCTTAATAAAAAAAAATAACTTCGTATAGCATACATTATACGAAGTTATACAATGCACTTTTTGAGTACAG	Production of reporter constructs
MalP4750	GCACATAATATGTAAAATAGTTATATGGGTACACAATATTTATGGTATATCTAAGTAAG	Production of reporter constructs
MalP4928	ACGCCAGCTGGCGAAAGG	ChIP assays
MalP4929	GGCCAATTCGCCCTATAGTGAGTCG	ChIP assays
MalP4969	GCAACCTTAAAAAGAAAAAAAGAGTACAAAC	Production of reporter constructs
MalP4970	CTCTTTTTTTCTTTTTAAGGTTGCATAACTTCGTATAATGTATGCTATACGAAGTTATGTACAGGTATTGTACGCC	Production of reporter constructs

Table S1. Oligonucleotide list related to Materials and Methods
